# Non-contact monitoring of the depth temperature profile for medical laser scanning technologies

**DOI:** 10.1038/s41598-020-77283-9

**Published:** 2020-11-20

**Authors:** Jure Kosir, Daniele Vella, Matija Jezersek

**Affiliations:** Faculty of Mechanical Engineering, University of Ljubljana, Askerceva 6, Ljubljana, Slovenia

**Keywords:** Biomedical engineering, Engineering

## Abstract

Medical treatments such as high-intensity focused ultrasound, hyperthermic laser lipolysis or radiofrequency are employed as a minimally invasive alternatives for targeted tissue therapies. The increased temperature of the tissue triggers various thermal effects and leads to an unavoidable damage. As targeted tissues are generally located below the surface, various approaches are utilized to prevent skin layers from overheating and irreparable thermal damages. These procedures are often accompanied by cooling systems and protective layers accounting for a non-trivial detection of the subsurface temperature peak. Here, we show a temperature peak estimation method based on infrared thermography recording of the surface temperature evolution coupled with a thermal-diffusion-based model and a time-dependent data matching algorithm. The performance of the newly developed method was further showcased by employing hyperthermic laser lipolysis on an ex-vivo porcine fat tissue. Deviations of the estimated peak temperature remained below 1 °C, as validated by simultaneous measurement of depth temperature field within the tissue. Reconstruction of the depth profile shows a good reproducibility of the real temperature distribution with a small deviation of the peak temperature position. A thermal camera in combination with the time-dependent matching bears the scope for non-contact monitoring of the depth temperature profile as fast as 30 s. The latest demand for miniaturization of thermal cameras provides the possibility to embed the model in portable thermal scanners or medical laser technologies for improving safety and efficiency.

## Introduction

Medical laser-based technologies move the trend toward the use of less invasive techniques in clinical protocols. Most performed aesthetic medical treatments are often accompanied by adverse effects, such as scaring, post-procedural pain, prolonged recovery time, and significant complications^1–3^. A lot of effort has been made searching for new minimally invasive techniques, including high-intensity focused ultrasound (HIFU), low-level laser therapy (LLLT), cryolipolysis, radio frequency (RF), and hyperthermic laser lipolysis (HLL)^3–5^. Some of those alternative tissue therapies, along with laser-induced thermotherapy (LITT)^6^, HLL and RF are hyperthermia-based. During HLL therapy, laser energy is transmitted and absorbed by adipocyte cells, leading to increased temperature of subcutaneous adipose tissue. Therefore, various thermal effects can be triggered, including hyperthermia (45 °C), coagulation (60 °C), and decomposition (thermal ablation) (100 °C)^7^. Tissue damages linearly depend on the heating time and exponentially on the temperature increase^8^, hence the effectiveness of the treatment heavily rely on the ability to closely monitor and field of the tissue^9^. Accurate monitoring of the established temperature field was described as ‘a crucial ingredient of any hyperthermia procedure’ by Christensen^10^, and an essential to widespread such procedures^11,12^.

Active control of surface temperature with a closed-loop control system was already demonstrated by utilizing a low spatial resolution infrared sensor for a laser system modulation^7,13^. The system is applicable to laser tissue welding, where peak temperature is located at the tissue surface.

However, targeted tissues are often located below the tissue surface (subcutaneous layer) and various techniques require protective layers or cooling systems to prevent upper layers of tissue from overheating^4,5,14^. Therefore, temperature peak shifts into the subcutaneous layer, and its active monitoring becomes inaccessible to medical practitioners. In this scenario, numerous approaches were proposed to monitor temperature field within the tissue, including insertion of thermocouples or fiber-optic sensors^15^, optoacoustic temperature monitoring^16,17^, magnetic resonance imaging (MRI)^18^, different optical methods^19,20^, impedance tomography^21^, and ultrasound^22,23^. Nevertheless, some of these methods are complex or either invasive, with low penetration or inadequate spatial resolution, and expensive.

Low penetration is a limiting factor for infrared thermography as well. This problem has been addressed by analyzing the temporal surface field characteristics of temperature, as demonstrated in several studies of thermoelasticity, hyperthermia therapy, and laser lipolysis^1,4,24,25^. Some of these studies aimed at specific therapy processes (e.g., laser-tissue interaction coupled with cooling) and required a tailoring physical model that somehow limits broader applicability and implementation.

Here, we present the application of infrared thermography to estimate the depth temperature profile (DTP) within the tissue. DTP estimation method was used in feasibility studies where a hyperthermic laser lipolysis (HLL) was performed on an ex-vivo porcine fat tissue. An initial arbitrary DTP generation function and thermal-diffusion-based model were coupled to generate the time-dependent database. The algorithm was further employed to correlate measured surface temperature evolution with the calculated solutions. As a result the DTP was reliably estimated with a small deviation from the real temperature field. Moreover, ex-vivo approach allowed proving the validity of the protocol by direct measurement of the reference DTP when the sample was split in two halves. In addition, we introduced an extension of the protocol to a more realistic in-vivo scenario.

## Methods

### Algorithm process flow

The DTP estimation method relies on a time-dependent surface temperature field (*T*_surf_(*t*)), that is measured during the clinical procedure when the laser and cooling system are turned off. During the active period, the temperature peak (*T*_max_) undergoes a shift below the tissue surface (*z*_max_), due to the joint effects of water evaporation from the surface^26^ and external air cooling^4,27^. The measuring time interval *T*_surf_ changes with a rate that mainly depends on the starting temperature distribution (internal gradient), environmental conditions and thermal properties of the tissue.

**Figure 1:**
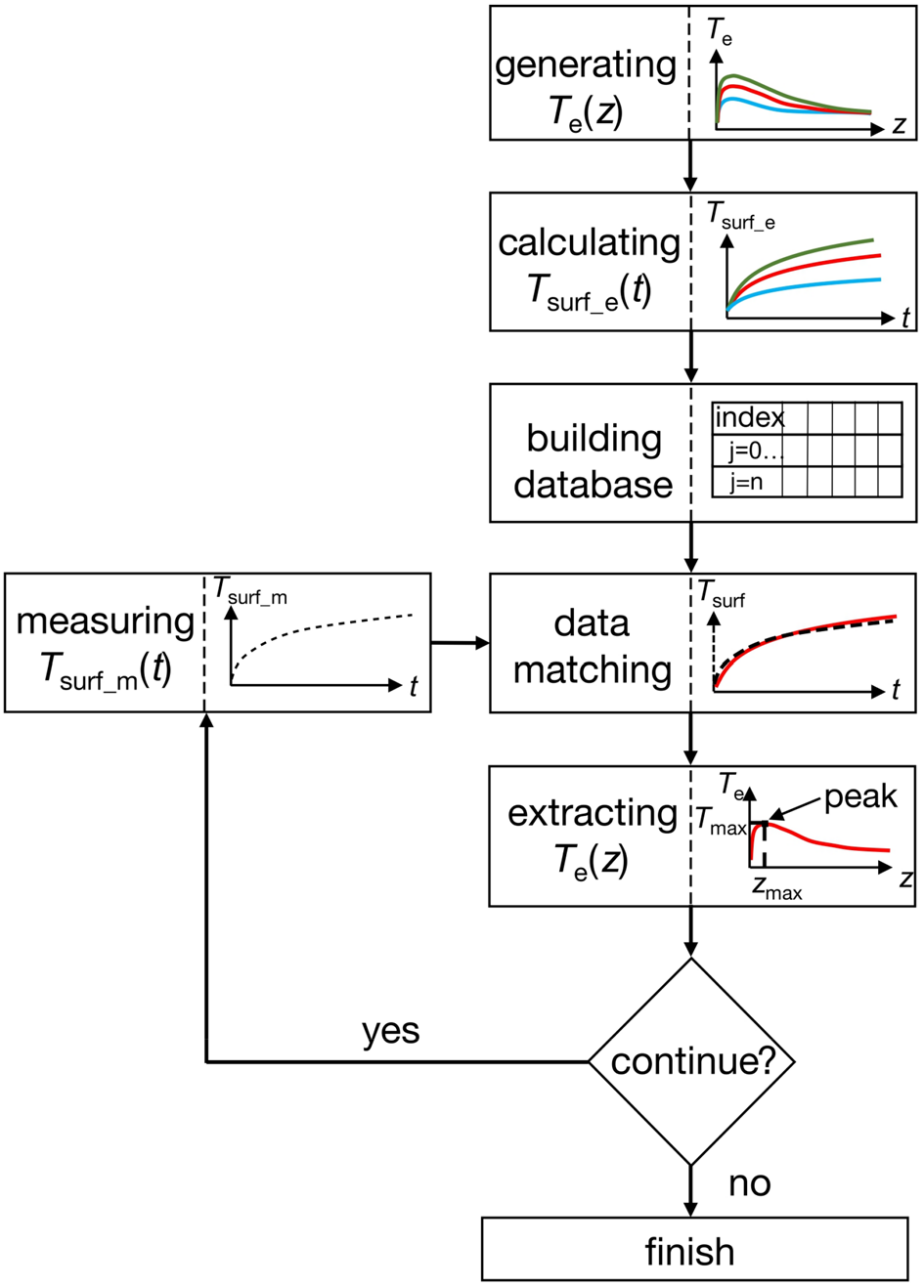
Block diagram of the depth temperature field estimation. Various generated T_e_(z) were used as an initial condition in the model for calculations of corresponding surface temperature evolutions T_surf_e_(t) to build the database. Measured T_surf_m_(t) was correlated with the database via time-dependent data matching process. Final T_e_(z) was obtained upon the closest fitting of T_surf_m_(t) and T_surf_e_(t).

The algorithm, presented in Fig. 1, was custom developed in C++ IDE (Code::Blocks, 16.01). It first calculates a group of possible temperature field distributions (*T*_e_(*z*)) and time evolutions that are clustered in a database. The thermal diffusion model calculates the time-dependent surface temperature (*T*_surf_e_(*t*)) for all *T*_e_(*z*) generated (see section “Consideration of the estimation method”). The thermal camera measures the evolution of the surface temperature (*T*_surf_m_(*t*)), which is correlated with an ensemble of calculated *T*_surf_e_(*t*) via the data matching process. The closest fit of *T*_surf_e_(*t*) enables selection of the correspondent *T*_e_(*z*). The *T*_e_(*z*) represents the final estimation of the measured temperature field within the tissue, denoted by *T*_m_(*z*).

### Sample preparation

We used a porcine fat tissue as it sufficiently exemplifies optical and thermal properties of a human tissue^28–30^. A homogeneous fatty tissue was collected approximately 48 h before experiments and was held at 4 °C. Prior to measurements, sample was thermalized at room temperature for two hours. The sample was shaped in an 80 × 100 mm^2^ and 34 mm thick plate. Right before measurements it was halved with a thin blade, resulting in two symmetrical halves, each measuring 80 × 50 mm^2^.

### Experimental setup and measurement protocol

**Figure 2:**
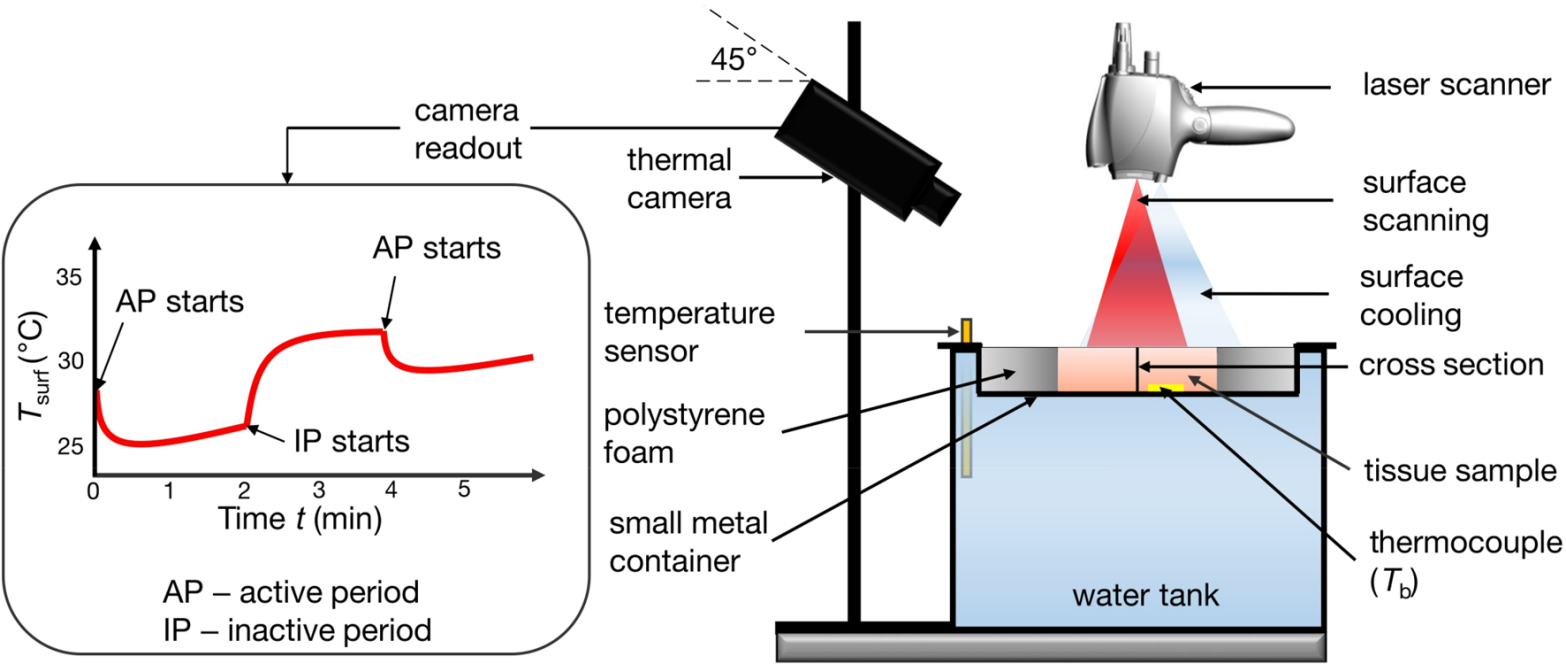
Schematics of the experimental setup. The sample surface was irradiated with a Nd:YAG laser system (1064 nm), and simultaneously forced-air cooled. The sample was in contact with a small metal container and partially submerged in a water tank to ensure a constant temperature at the bottom side T_b_, measured with a thermocouple. Thermal camera readout shows the evolution of the T_surf_(t) during both active (AP) and inactive (IP) periods.

**Figure 3:**
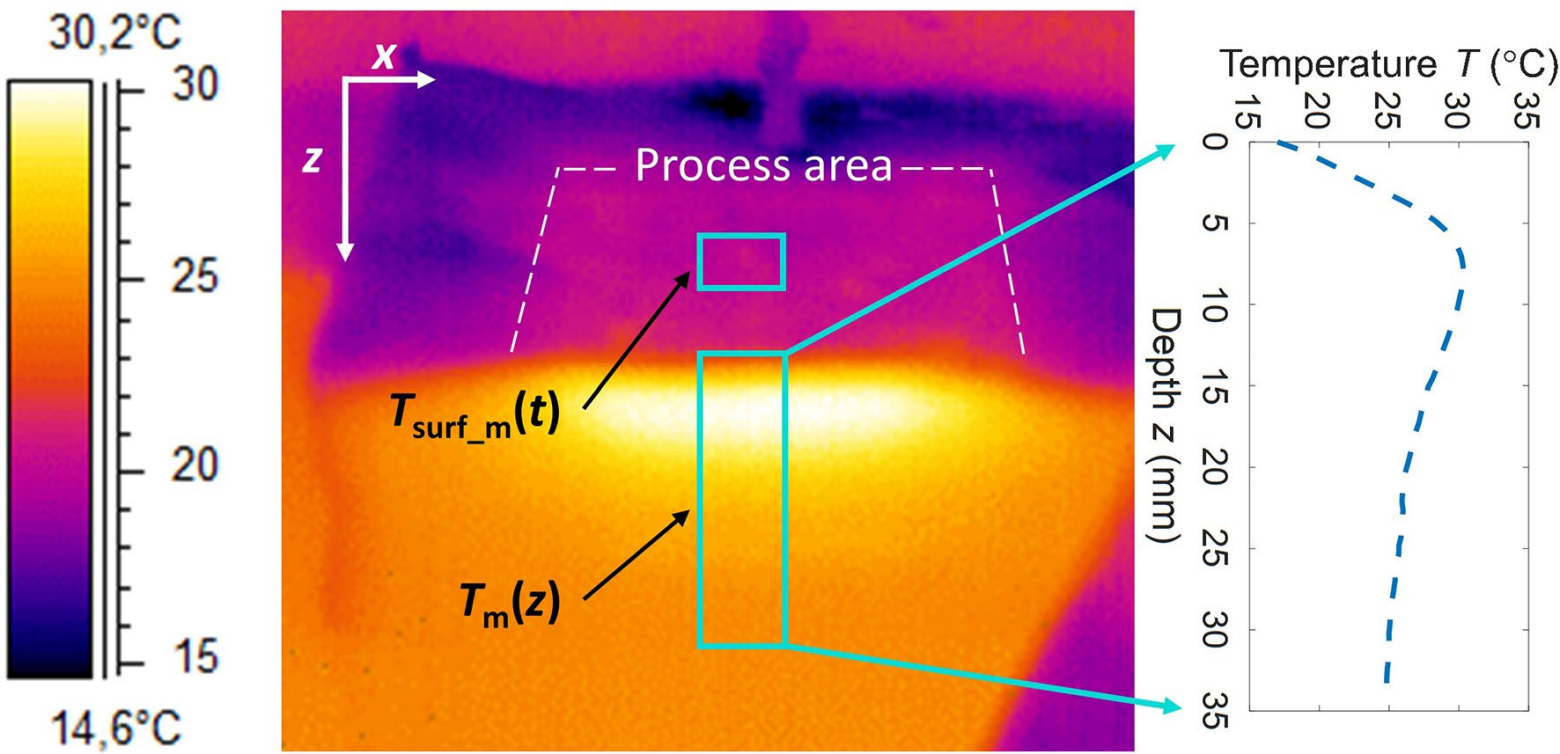
Thermal image of one symmetrical halve recorded immediately after its separation. T_m_(z) distribution (right panel) extracted from the cross-sectional area (light blue rectangle) by averaging temperature data point in the x-direction. A portion of the outlined process area (light blue rectangle) was used to record the surface temperature T_surf_m_(t) evolution.

Experimental setup was designed and assembled in a way that it best replicated the realistic HLL procedure. The setup included a 1064 nm Nd:YAG laser source (SP Dynamis, Fotona, Slovenia) with a high-speed laser scanner (S-11, L-runner mode, Fotona, Slovenia), forced-air cooling system (Cryo 6, Zimmer, Germany), and a thermal camera (ThermaCAM P60, FLIR, 7.5–13 µm) as shown in Fig. 2. System effectively elevates the temperature of targeted tissue, while forced-air cooling maintains *T*_surf_ below the thermal damage threshold level. The HLL procedure can be divided into two periods—the active period (laser irradiation and cooling system turned on), and the inactive period, when both systems are turned off (thermal relaxation of tissue). The effect of induced temperature gradient (indicated in Fig. 3) can be observed as a *T*_surf_(*t*) rise during the inactive period, as schematically shown on the left side in Fig. 2.

Both sample halves were inserted in a small metal container (approx. 3.5 l). The remaining space was filled with polystyrene foam, limiting undesired tissue-air interactions and providing additional tightness to the overall cross-sectional area. A small metal container was only partially submerged in a water tank to prevent any water leakages. With good thermal conductivity of the small metal container and a large volume of the water tank (approx. 20 l), the temperatures at the bottom of the tissue sample *T*_b_ were stable during the measurements. The temperature of the water in the large volume tank was at a room temperature (25 °C). We intentionally maintained water at this temperature in order to achieve smaller cooling rate at the cross-sectional area when halves were separated. This allowed to minimize the error while obtaining the reference measurement.

Real-time monitoring of the *T*_b_ was realized with a thermocouple sensor (d = 1.2 mm) inserted into the bottom side of the tissue sample beforehand. For temperature field recording, the thermal camera was mounted on a fixed holder at an angle of 45°, relatively to the sample surface. Such placement allowed simultaneous recording of the spatial temperature field *T*_m_(*z*) within the tissue (see cross-section in Fig. 3), and *T*_surf_m_(*t*).

Prior to each measurement a paper tissue was used to dry the surface of the sample. Laser irradiance and forced-air cooling were simultaneously applied to the upper surface of the tissue sample, starting the active period. After the active period (120 s), both systems were turned off, flagging the inactive, thermal relaxation period. This was done by stopping the laser irradiation and immediate turning the scanner head, including cooling nozzle, away from the sample. Immediately after, one halve of sample was pulled away from the other halve which was fixed to the edge of large tank. Thermal camera was already recording the sample and in post-processing phase we selected the first image, where the entire cross-section of the sample was visible. This image was used as a reference DTP. The average time delay of this procedure was 0.64 s, which we measured with thermal camera. Corresponding maximal temperature drop of DTP was approximately 0.13 °C. Simultaneously, sample halves were separated, and cross-sectional temperature distribution was recorded with a thermal camera. Additionally, the *T*_surf_m_(*t*) and environmental temperature were recorded. This process represented one full measurement, resulting in *T*_m_(*z*) and its belonging *T*_surf_m_(*t*) evolution. For all measurements, the image, recorded right at the halves separation, was identified and analyzed in ThermaCam Researcher Pro 2.10 software (see Fig. 3) to determine the measured *T*_m_(*z*).

Laser irradiation was performed with an average intensity of *I* = 1.2 W/cm^2^. Homogeneous heating of tissue surface (see Fig. 3) was achieved with consecutive scans. The laser scanner generated a laser spot of 11 mm in diameter at the sample surface with a top-hat beam profile. One scan lasted 1.04 s and required 27 pulses to cover a surface area of 54 × 57 mm^2^. Repetition rate was 25 Hz and laser pulse duration was 600 µs.

The sample surface was cooled with a forced-air cooling system, which maintains air temperature at − 30 °C. During the active period, the outlet cooling nozzle was at a constant distance of 20 cm, as it was integrated with the laser scanner head. Because we only seek the initial *T*_m_(*z*), immediately after the active period, forced-air cooling was excluded from the mathematical model, and cooling characteristics were not extensively measured in this study. However, reported by Milanic et al., the cooling air temperature was measured substantially higher at the sample surface (approximately 10 °C)^4^.

Measurements were designed to test the flexibility of the estimation method in a way that different distributions of *T*_m_(*z*) within the tissue were achieved, by systematically changing procedure parameters. Although laser irradiation and cooling both highly participate in *T*_m_(*z*) distribution, only the cooling was modified in our experiment. Average intensity of the laser beam at the sample surface remained constant for all measurements.

**Figure 4:**
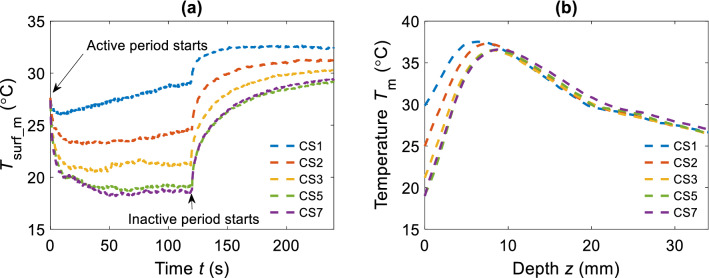
Surface temperature and DTP measured at different cooling settings (CS). (**a**) Surface temperature T_surf_m_(t) recorded during a full protocol. (**b**) Corresponding T_m_(z) measured at the start of the inactive period.

By modifying the cooling system fan speed, air velocity at the exit nozzle changed, resulting in a variety of depth temperature profiles. In our experiment we used fan speeds of 1, 2, 3, 5 and 7 to modify cooling settings (CS). The correspondent *h*_t_ was estimated prior, ranging from 60 to 125 W/m^2^ K. Starting with the lowest CS1 (*h*_t_ ~ 60 W/m^2^ K), the cooling setting was successively increased to get a set of five different *T*_m_(*z*) and *T*_surf_m_(*t*), representing one full cycle of measurements. Between each measurement, the tissue sample was thermalized by natural convection for 30 min. A complete cycle of *T*_surf_m_(*t*) is shown in Fig. 4a, and the corresponding *T*_m_(*z*) in Fig. 4b. The same cycle was repeated three times.

## Results and discussion

### Estimation of the subsurface temperature peak

**Figure 5:**
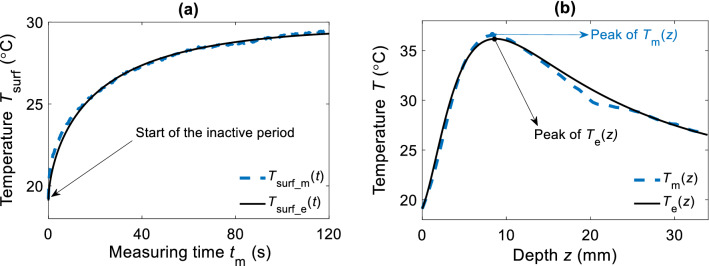
Fitting of T_surf_m_(t) and prediction of the T_m_(z). (**a**) Measured surface temperature T_surf_m_(t) (blue dashed line) and its fit (black solid line) T_surf_e_(t). (**b**) Comparison between measured distribution (blue dashed line) T_m_(z) and the predicted distribution T_e_(z) (black solid line).

A wide set of temperature fields *T*_surf_m_(*t*) and *T*_m_(*z*) were recorded to test the flexibility of the estimation method (see measured *T*_m_(*z*) in Supplementary Fig. S1a–c). For all of the measurements *T*_surf_m_(*t*) were used as an input to the matching algorithm. As a result, the corresponding temperature fields on the surface and within the tissue were picked from the ensemble’s database. Figure 5a,b show respectively the best fit to the measured *T*_surf_m_(*t*) and the estimated *T*_e_(*z*) that best reproduced measured temperature field *T*_m_(*z*).

Accuracy of the estimation method was evaluated (MATLAB 2020a, Mathworks Inc., Natick, MA) comparing *T*_e_(*z*) and *T*_m_(*z*) in terms of differential peak position Δ*z*_max_ and peak temperatures Δ*T*_max_. Average ${\Delta }z_{{{\text{max}}}}$, and average ${\Delta }T_{{{\text{max}}}}$ over three consecutive measurement cycles, show a small deviation from the peak temperature and its position. Using the entire recorded *T*_surf_m_(*t*) evolution (120 s), ${\Delta }z_{{{\text{max}}}}$, and ${\Delta }T_{{{\text{max}}}}$ were 0.012 ± 0.3 mm, and 0.01 ± 0.25 °C respectively.

Three cycles of measurement were performed, utilizing sweeping CS (from CS1 to CS7) for each cycle. In all the cycles *z*_max_ shifts deeper into the fat tissue with increasing CS (see Supplementary Fig. S1a–c).

For each cooling rate, the average of *z*_max_ shows two different trends. A substantial shift, approximately 12–15% at lowest CS (*h*_t_ in range of 60–80 W/m^2^ K), and a less remarkable change at higher CS (*h*_t_ > 100 W/m^2^ K), ~ 2% (see Fig. S1a–c). This discrepancy may be due to the decrease of the relative difference between two successive *h*_t_. However, during the first cycle the peak temperature *T*_max_ increased along with CS (see Supplementary Fig. S1a). This indicates that the sample was not sufficiently thermalized at room temperature (RT) after being at 4 °C prior to measurements. Instead, second cycle reveals almost constant peak temperature (~ 35 °C) despite increasing CS, as sample was longer thermalized at RT (see Supplementary Fig. S1b). In the third cycle the innermost temperature was expected to be more homogenous and closer to RT. In fact, *T*_max_ decreases with higher CS (see Supplementary Fig. S1c) as theoretically predicted^4^. Likewise *z*_max_, the *T*_max_ undergoes a clear drop that becomes negligible at higher CS. As previously observed in a theoretical work by Milanic et al., our finding shows comparable trend for *z*_max_ dependency on *h*_t_ (see Supplementary Fig. S1d). Measurements which best represented theoretical prediction are showed in Fig. 4b, where both *T*_max_ and *z*_max_ get respectively lower and shift deeper with increasing CS (third cycle).

**Figure 6:**
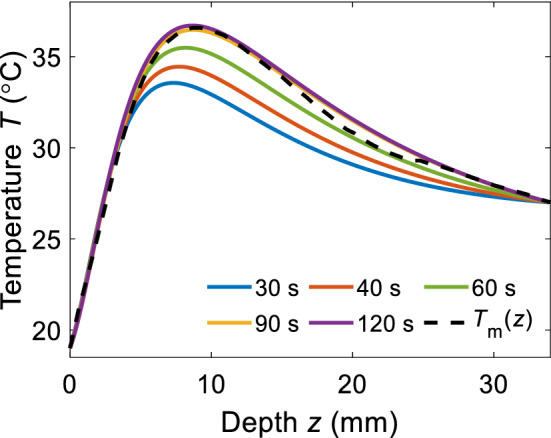
T_e_(z) evolution at different measuring time t_m_. Accuracy of the estimation approaches the real data (black dashed line) with increasing t_m_.

We now consider the measuring time parameter *t*_m_, that is a temporal window segment of the entire recorded *T*_surf_m_(*t*). Choosing the right duration of *t*_m_ is crucial because it can compromise the results of the matching algorithm or extend the measuring period on the other hand. An estimation accuracy improvement with progressing *t*_m_ in the data matching process is shown in Fig. 6. Best prediction of the dynamics is obtained for measuring period *t*_m_ ≥ 90 s, indicating a convergence of the estimation accuracy.

**Figure 7:**
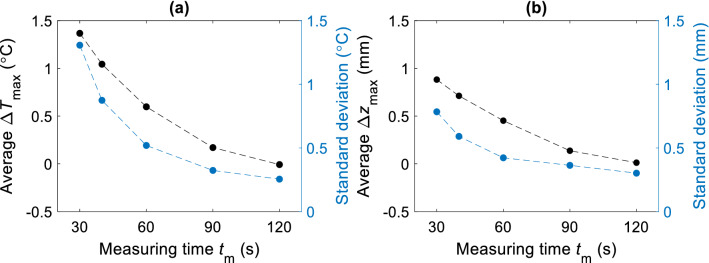
Estimators $\Delta T\mathrm{_{max}}$ and $\Delta z\mathrm{_{max}}$ at different t_m_. (**a**) Average $\Delta T_{\mathrm{max}}$ (black dots) and its standard deviation in absolute values (blue dots, right axis). (**b**) Average $\Delta z_{\mathrm{max}}$ and its standard deviation. In both cases the differential error becomes smaller and converges on a longer time scale.

By changing *t*_m_ in the data matching process, we evaluated accuracy improvement with ${\Delta }z_{{{\text{max}}}}$ and ${\Delta }T_{{{\text{max}}}}$ parameters for all three cycles of measurements. Both parameters, as a function of duration of *t*_m_ are shown in Fig. 7a,b. Results show a convergence of estimation errors to zero as *t*_m_ duration increases. Likewise, the average standard deviation decreases ~ 50% by doubling the duration of *t*_m_, thus increasing overall estimation confidence of *T*_m_(*z*).

**Figure 8:**
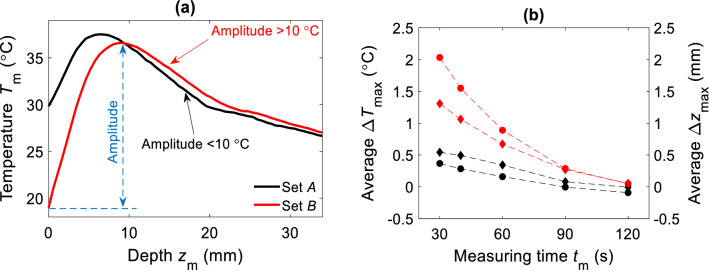
The dependency of the estimation accuracy on the amplitude of the measured T_m_(z). (**a**) Illustration of two types of measurement sets. Set A included T_m_(z) with amplitudes smaller than 10 °C (black curve), and set B included T_m_(z) with amplitudes bigger than 10 °C (red curve). (**b**) Estimation accuracy at different t_m_ for both data sets. Data points marked with circles are referred to $\Delta T_{\mathrm{max}}$ (black color for set A, red color for set B). Data points marked with diamonds are referred to $\Delta z_{\mathrm{max}}$. At lower cooling rate (set A), the deviation $\Delta T_{\mathrm{max}}$ and $\Delta z_{\mathrm{max}}$ are kept respectively below 0.5 °C and 0.5 mm.

Moreover, *t*_m_ dependency becomes more evident when different cooling settings affect the amplitude of the *T*_m_(*z*). As mentioned before, we recorded a wide set of distributed *T*_m_(*z*) with different amplitudes, ranging from 3.5 to 17.5 °C. We separated *T*_m_(*z*) into two data sets that we called set *A* and *B*, as depicted in Fig. 8a. Set *A* included *T*_m_(*z*) with amplitudes smaller than 10 °C (mostly obtained with CS1 and CS2), and remaining *T*_m_(*z*) with amplitudes bigger than 10 °C (obtained with CS3, CS5 and CS7) were included in set *B*. Estimators ${\Delta }T_{{{\text{max}}}}$ and ${\Delta }z_{{{\text{max}}}}$ are showed in Fig. 8b for both sets.

For set *A*, we observed a weak dependency of the estimators on the *t*_m_ duration. In contrary, for set *B*, a noticeable dependency of ${\Delta }T_{{{\text{max}}}}$ and ${\Delta }z_{{{\text{max}}}}$ on *t*_m_ can be seen, encompassing for an error convergence at longer *t*_m_. This could be explained by considering that temperature of the tissue, achieved during the cooling, delays the temporal window for which the thermodynamic equilibrium at the surface is reached. Hence, the time constant of *T*_surf_m_(*t*) dynamics, during the inactive period, influences the optimal estimation of the inside temperature profile. Fast dynamics observed for set *A* (see curves in Fig. 4a for comparison) will require shorter *t*_m_ for a reasonable estimation of *T*_m_(*z*) (see Fig. 8b). In this case, *t*_m_ has limited influence on the out coming result, meaning that the inactive period can be as short as 30 s.

### Consideration of the estimation method

Reverse analytical solution of heat diffusion that leads to an estimation of the initial distribution of the temperature field is severely ill-posed^31–33^. A small variation of the measured thermal signal can significantly affect the prediction of the inner temperature distribution. Many complex analytical and numerical approaches^31,33^, sometimes implemented on an artificial neural network^25^, have been used to predict the depth temperature profiles in tissues such as skin and blood vessels or tumor-mimicking inclusion^34^. For instance, thermal response of the skin has been measured by pulsed photothermal radiometry and reconstructed using a combination of numerical methods^35^. Likewise, a remarkable effort has been done to develop reconstructive algorithm for predicting increase in temperature during HIFU treatments^33^. One way to do that is using finite element solution of the bioheat transfer equation combined with Tikhonov regularization method^33^, or estimating the apparent time shifts in the ultrasound radiofrequency signal^34^. Our assumption for modeling considers a homogeneous tissue, since the adipose layer of the sample was homogenously distributed and represents a simplification in comparison with the approaches mentioned above.

Thermal stimulation, induced with HIFU requires high spatial and temporal resolution, but above the boiling point (coagulation regime) high temperature inaccuracy is acceptable (approximately 2–3 °C)^22^. On the other hand, hyperthermic treatments with smooth thermal gradients and larger thermal mass tolerate lower spatial (2–3 mm) and temporal resolution^22^. However, the required temperature precision is usually less than 1 °C^22^. Our results show inaccuracies of the temperature peak estimation below 1 °C within a temperature sampling time comparable to the one used in HIFU^34^, and spatial inaccuracy of peak temperature below 1 mm. Similar accuracy with a deviation of 10% on the relative temperature was also achieved in photothermal therapy by volumetric reconstruction of the optoacoustic signal^16^.

However, none of these methods can be successfully employed when a larger area is processed and fast non-contact monitoring of DTP is required. For instance, HLL procedure usually employs a laser scanning system and an optoacoustic reconstruction method would require an ultrasound transducer with a volumetric dimension much larger compared to a single spot application. Photoacoustic thermal monitoring and 3D reconstruction could be more suitable for localized treatment areas, such as minimally invasive surgery and even tumor or vessel ablation. Another advantage of the proposed method is its non-contact nature, which avoids the insertion of a thermocouple within the skin that can induce a discrepancy between the probed area and the temperature of the surrounding tissue^15,22^.

We now describe in detail the depth temperature profile (DTP) estimation method and compare the physical parameters used in the temperature estimation with previous work. The model considers the temporal evolution of the surface temperature in a homogenous tissue. The time-dependent matching process is supported by generated database and overcomes the reversibility problem of a heat equation, as already mentioned. Our approach starts with an introduction of an arbitrary field distribution *T*_e_(*z*) that approximates a realistic DTP, see Eq. (1):

1 $$T_{{\text{e}}} (z) = (T_{{{\text{surf}}}} - 1) + (z + 1)^{{\frac{{\text{B}}}{{{\text{C}}\left( {z + 1} \right) + {\text{A}}}}}} - {\text{D}} \cdot (z + 1)$$

The dimensionless parameters *A*, *B* and *C* define distribution characteristics of the generated *T*_e_(*z*) temperature field, parameter *D* [°C/mm] defines the correction for bulk temperature deeper in the tissue. For in-vivo studies, bulk temperature can be set to 36 °C. By modifying those parameters, temperature distribution *T*_e_(*z*) can be varied in order to adjust the subsurface temperature peak, surface temperature, or bulk temperature (see Supplementary information, section Description of arbitrary function parameters). Resolution of the database is given by appropriately choosing the set of parameters above described, taking into account the tradeoff between accuracy and computational time. Similar DTP was obtained from ultrasound signal generated in a tumor mimicking inclusion filled with plasmonic nanoparticle (tissue phantom and an ex-vivo porcine muscles)^34^, and in a study of temperature-dependent blood perfusion^36^.

The absence of laser heating and forced-air cooling during the inactive period enables the use of basic laws of transient heat transfer. From generalized Fourier’s equation^37^, temperature field evolution can be written as follows:

2 $$\rho c_{p} \frac{\partial T(z,t)}{{\partial t}} = k\nabla^{2} T(z,t)$$

where $\rho$ stands for mass density, $c_{p}$ for specific heat, and $k$ for thermal conductivity of the observed sample. Thermal properties of simulated fat tissue were $\rho = 860$ kg/m^3^, $c_{{\text{p}}} = 2870$ J/kgK, and $k = 0.23$ W/mK^1, 38^.

Field distributions *T*_e_(*z*), generated with Eq. (1), were used as initial condition for time-dependent solutions, calculated by Eq. (2). The interface boundary condition, applied at the air-tissue boundary governs the heat losses, due to natural convection, and body radiation as follows:

3 $$- k\frac{{\partial T_{{{\text{surf}}}} (t)}}{{\partial {\text{z}}}} = h_{{{\text{NC}}}} (T_{{{\text{air}}}} - T_{{{\text{surf}}}} (t)) + \sigma \varepsilon (T_{{{\text{env}}}}^{4} - T_{{{\text{surf}}}} (t)^{4} )$$

$h_{{{\text{NC}}}}$ represents heat transfer coefficient ($h_{{\text{NC}}} =$ 13 W/m^2^ K), $\sigma$ the Stefan–Boltzmann constant ($\sigma = 5.67 \times 10^{ - 8}$ W/m^2^ K^4^), and $\varepsilon$ sample emissivity ($\varepsilon = 0.98$)^37,39^. *T*_surf_ stands for temperature at the surface of the sample, *T*_air_ for temperature of the air in contact with the sample surface, and *T*_env_ for surrounding temperature.

In the calculations, we used *k* in range from 0.17 to 0.32 W/mK. Best results were obtained for $k = 0.23$ W/mK, which correspond very well with an average $k = 0.233 \pm 0.006$ W/mK, measured for subcutaneous pig fat without the skin^38^. Moreover, we chose $h_{{{\text{NC}}}} = 13$, which fits well within the range of common values between 10 and 15 W/m^2^ K used in several studies^1,4,40,41^.

The evaporation of water from the surface was not explicitly considered, since evaporative heat losses were not a dominant factor. In fact, surface of the sample did not contain visible moisture, and *T*_surf_ remained way below 60 °C. Above this critical temperature water evaporation from the surface outweighs the other surface loss terms (convection and radiation), and leads to denaturation processes such as water-complex dissociation or structural phase changes^26,42^. Here, the evaporative heat losses were not neglected, but attributed to the convective heat transfer coefficient (*h*_NC_). This represents a common approach that has been adopted in many studies^1, 27,43^. Moreover, no visible moisture accounts for less than 6% of maximal evaporative heat loss and studies estimate that the contribution of evaporative heat losses from dry skin is commonly less than 10% of the total convective term^44,45^.

We analyzed the possibility to extend the method to an in-vivo procedures by providing a more complex in-vivo tissue model (IVM) (see detailed description in Supplementary information, section In-vivo tissue model (IVM)) combined with the arbitrary function (Eq. (1)). In an in-vivo experiment, the estimated DTP validation is challenging. Therefore, we considered a comparable in-vivo study by Milanic et al.^27^, wherein an estimation of DTP_MCML_ was obtained by weighted Monte Carlo photon multi-layer procedure. We adopted (from the same study^27^) the surface temperature evolution *T*_surf_in-vivo_(*t*), measured during the inactive period, and used it as an input to the data matching function (see Fig. S2a). Estimated in-vivo DTP_INV_ was successively compared to the DTP_MCML_ (see Fig. S2b), adopted from the same study^27^. From the comparison of the two temperature depth profiles we estimated a deviation of the peak temperature and position respectively, Δ*T*_max_ = 0.5 °C and Δ*z*_max_ = 0.4 mm (see Supplementary information, section Estimation of the DTP from in-vivo measurement, Fig. S2b). Moreover, the shape of the estimated DTP_INV_ reveals great similarity to the DTP_MCML_, which is well known to be the most used theoretical approach in this research field.

The presented non-contact method lays the groundwork for monitoring the depth temperature profile and for calibration of the laser parameters. This concept could be employed during the inactive period, in a long laser treatment, to better address the efficiency of the therapy in the next active cycle. By further research, we speculate a possibility to develop a feed-back control loop to adjust laser parameters and cooling system, based on a real-time measurement of the surface temperature and a light-tissue interaction model. This could lead to a prediction of the DTP profile during laser irradiation and correct calibration of the laser parameters at the beginning of clinical protocol.

## Conclusion

Monitoring of the inner temperature profile is not trivial and becomes relevant in medical laser-based therapies. Here, we developed a non-contact temperature estimation method by combining the flexibility of thermography with a novel time-dependent data matching approach. The method was experimentally carried on an ex-vivo porcine fat tissue which allowed for measurements of cross-sectional DTP and reliable validation of the algorithm. Our results show an accurate prediction of DTP by monitoring the evolution of the surface temperature. Average ${\Delta }T_{{{\text{max}}}}$ and ${\Delta }z_{{{\text{max}}}}$ at different measuring time *t*_m_ show a small deviation from the measured temperature profiles. Reasonable estimation accuracy of 0.6 ± 0.5 °C (Δ*T*_max_) and 0.45 ± 0.4 mm (Δ*z*_max_) can be achieved with measuring time (*t*_m_) of 60 s. However, the dependency of the DTP estimation on *t*_m_ becomes almost negligible at the lowest cooling settings and ensures a good estimation as fast as *t*_m_ = 30 s. Moreover, we provide a further advanced tissue model that shows a good reproducibility of the in-vivo *T*_surf_(*t*) and the temperature distribution within a complex tissue. Therefore, the protocol paves the way for its extension to a clinical process that requires incremental heating, interrupted by inactive periods. In this temporal window the method could provide an evaluation of the DTP and calibration of optimal laser parameters for the next active period. Overall, our approach avoids necessity to specify a heating or cooling source and may support practitioners to appropriately calibrate parameters of medical device.

## Supplementary information

Supplementary Figure S1.

Supplementary Figure S2.

Supplementary Information.

## Data Availability

The data that support the findings of this study are available from the corresponding author upon reasonable request.
