# Supplementary material for: Non-contact monitoring of the depth temperature profile for medical laser scanning technologies
Source: Sci Rep. 2020 Nov 20;10:20242. doi: 10.1038/s41598-020-77283-9 (PMC7679450; doi:10.1038/s41598-020-77283-9)
Supplement: Supplementary file 3 — Supplementary Information. [file 41598_2020_77283_MOESM3_ESM.docx]

**Non-contact monitoring of the depth temperature profile for medical laser scanning technologies**

**AUTHORS: Kosir Jure^1*^, Vella Daniele^1^, Jezersek Matija^1^**

*^1^ Faculty of Mechanical Engineering, University of Ljubljana, Askerceva 6, Ljubljana, Slovenia*

**Supplementary information**

Supplementary figure S1. Measurements of depth temperature profiles. For all three cycles measurement z_max_ shifts deeper into the tissue when cooling settings CS were raised from lowest to highest. (a) The sample was not sufficiently thermalized at room temperature (RT), as a result the peak temperature T_max_ increases its intensity. (b) Peak temperature T_max_ remained relatively constant, as sample was more thermalized at RT. (c) Highest thermalization of the sample at RT, T_max_ decreases its intensity and shifts deeper with increasing CS, as predicted in theory^1^. (d) The penetration depth of the peak temperature z_max_ as a function of h_t_.

**Description of arbitrary function parameters**

In our case, parameter *B* was set to a constant value of 7. Parameters *A* and *C* were incrementally increased within a control flow statement (for-loop). The outer loop incremented the parameter *C* from 0.1 to 2 and the inner loop incremented parameter *A* from 0 to 15. Both parameters were incremented by a step of 0.1. By manipulating parameters *A* and *C* of the function, the bulk temperature *T*_bulk_ (at *z*=34 mm) also changed, whereas it had to be constant for all the generated temperature distributions *T*_e_(*z*). This effect on *T*_bulk_ was corrected for each *T*_e_(*z*) generated by introducing a parameter *D* [°C/mm], calculated as:

|  | $D=\frac{T_{bulk\_i}- T_{bulk\_f}}{z_{s}}$ | (S1) |
| --- | --- | --- |
|  |  |  |

Where *T*_bulk_i_ stands for the bulk temperature after the parameters *A* and *C* were modified, *T*_bulk_f_ for desired bulk temperature, and *z*_s_ for the thickness of the sample.

**Estimation of DTP from in-vivo measurement**

To validate the core principle of estimation method, we employed a mathematical model that best described homogeneous tissue used in validation experiments. However, considering an in-vivo procedure, the estimation of the depth temperature profile (DTP) becomes a challenge. Hence, we adopted an in-vivo study, which best corresponds with our experimental conditions of laser-tissue interaction. In a study by Milanic et al.^2^, an estimation of DTP_MCML_ was obtained by weighted Monte Carlo photon multi-layer procedure. We adopted the corresponding surface temperature response *T*_surf_in-vivo_(*t*), measured during the inactive period (refer to Milanic et al.^2^, Fig. 4a), and employed it as an input to our estimation algorithm. The mathematical model, used in our validation experiments, was further advanced to a more complex in-vivo model (IVM), whereas the same arbitrary function (equation (1)) was used to generate *T*_e_(*z*)’s. The IVM (see subsection In-vivo tissue model (IVM), equations (S2)–(S7)) was used to recalculate *T*_surf_e_(*t*). The environmental conditions, and tissue parameters were used as reported in this study^2^ and are listed in Table S1.

Combining the algorithm presented in Fig. 1 (of the main text) with *T*_surf_in-vivo_(*t*), the in-vivo DTP_INV_ was estimated and compared to the DTP_MCML_. Figure S2a shows the *T*_surf_e_(t) that best fits to the real data.

The outcome of the estimation is presented in Fig. S2b. In this case the deviations between DTP_MCML_ and DTP_INV_ can be misleading, as both depth temperature profiles represent an estimation of the actual temperature distribution within the tissue. However, the shape of the estimated DTP_inv90s_ (at *t*_m_ = 90s ) reveales great similarity to MCML approach (Fig. S2b), which is well known to be the most used theoretical approach in this research field. From the comparison of the two DTPs we estimated a deviation of the peak temperature and position respectively, Δ*T*_max_= 0.5 °C and Δ*z*_max_= 0.4 mm.

In Fig. S2b we include an estimation of DTP_INV30s_, where a shorter measuring time *t*_m_ of 30 s was used in the data matching function. Comparing it with DTP_MCML_, deviation in the peak was Δ*T*_max_= 0.9 °C and Δ*z*_max_= 0.1 mm.


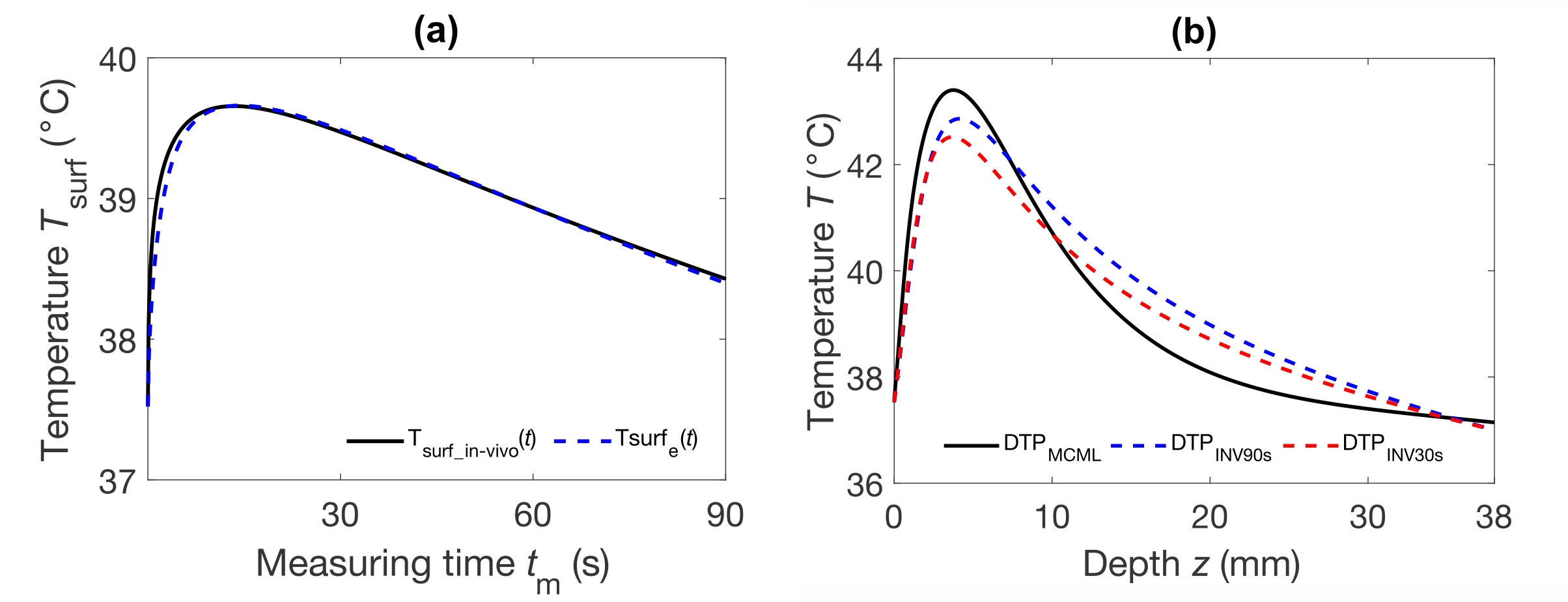


Supplementary figure S2. (a) Fitting of measured T_surf_in-vivo_(t) to a database calculated with IVM, using measuring time of 90 s. (b) Estimated DTP_INV90s_ and DTP_INV30s_ compared to the DTP_MCML_.

**In-vivo tissue model (IVM)**

For estimation of DTP in case of in-vivo measurement, we present two-layer tissue model with two planar homogeneous layers representing skin (epidermis and dermis) and fat tissue. In a living tissue, a blood perfusion also attributes to overall heat exchange^3^, hence this effect was introduced to the 1D equation for calculations of temperature field *T*(*z*,*t*) evolution. The 1D bioheat-diffusion equation now reads as follows^4^:

| $\rho c_{p}\frac{\partial T(z,t)}{\partial t}=k\nabla^{2}T(z,t)-F(T(z,t))\cdot c_{p,b}\cdot\omega_{b}\cdot\left[T(z,t)-T_{b}\right]$ | (S2) |
| --- | --- |

Where$\rho$ stands for mass density, $c_{p}$ for specific heat of tissue, and *k* for thermal conductivity. In a blood perfusion term the $c_{p,b}$ represents specific heat of blood, $\omega_{b}$ blood perfusion rate and *T*_b_ blood temperature. As the response of the vascular system to heat is temperature dependent below a certain threshold (*T*_F_), and it can be modeled with a temperature dependent scaling function, introduced by Drizdal et al. ^5^:

| $F\left( T \right)=\left\{ \begin{aligned} , \\ ,\mathrm{otherwise} \end{aligned} \right.$ | (S3) |
| --- | --- |

Where $\alpha$ accounts for the temperature depenceny of the blood perfusion rate, and $\beta$ represents the steepness of the blood flow change (values listed in Table S1). In study of Milanic et al. ^2^, the interface boundary condition applied at the air–tissue boundary was modified, as radiative heat losses were attributed to heat-transfer coefficient *h_T_* (10 W/m^2^K). Air temperature *T*_air_ was 21 °C. The boundary condition now reads as follows:

| $-k_{s}\frac{\partial T_{surf}(t)}{\partial z}=h_{T}(T_{air}-T_{surf}(t))$ | (S4) |
| --- | --- |

At the skin-fat interface a boundary condition must also be considered. Therefore, we adopted a “conductivity matching condition” approach for finite difference scheme calculations of heat transfer problem, where a matching point lies at an interface^6^. Geometrical properties of each layer are listed in Table S1. When computing equation (S2), the following condition was applied at the boundary point:

| $D_{b}\frac{\partial^{2}T_{b}}{\partial x^{2}}=\frac{1}{2}\cdot\left(D_{s}\frac{\partial^{2}T_{b-}}{\partial x^{2}}+D_{f}\frac{\partial^{2}T_{b+}}{\partial x^{2}}\right)$ | (S5) |
| --- | --- |

Where the *D* represents diffusion of particular layer. Subscript “b” stands for boundary, “s” for skin, and “f” for fat. The two terms in equation (S5), are given by equation (S6) and (S7):

| $\frac{\partial^{2}T_{b-}}{\partial x^{2}}=\frac{(2k_{s}+3k_{f})T_{b-2}-(2k_{s}+6k_{f})T_{b-1}+4k_{f}T_{b+1}-k_{f}T_{b+2}}{3(k_{s}+k_{f})\cdot\Delta x^{2}}$ | (S6) |
| --- | --- |
| $\frac{\partial^{2}T_{b+}}{\partial x^{2}}=\frac{-k_{s}T_{b-2}+4k_{s}T_{b-1}-(6k_{s}+2k_{f})T_{b+1}+(3k_{s}+2k_{f})T_{b+2}}{3(k_{s}+k_{f})\cdot\Delta x^{2}}$ | (S7) |

Where a “b-” in subscript denotes a region left of the boundary, and “b+” the region on the right of the boundary. Δ*x* represents a distance between the two points.

For each layer, a thermal diffusivity *D* ($k/\rho c$) was calculated with parameters specific for each layer: $c_{\mathrm{skin}}$=3500 (J/kgK), $c_{\mathrm{fat}}$=2870 (J/kgK), $\rho_{\mathrm{skin}}$=1090 (kg/m^3^), $\rho_{\mathrm{fat}}$=860 (kg/m^3^). For our calculations, the specific heat of blood $c_{p,b}$=3800 (J/kgK)^5, 7^ and *T*_b_= 37 °C were used ^2^.

Supplementary table S1: Thermal and blood perfusion properties for in-vivo tissue model^2^.

| Layer | *D* (m^2^/s) | *k* (W/m^2^K) | d (mm) | $\alpha$ | $\beta$ (°C^2^) | *T*_F_ (°C) | $\omega_{b}$ (kg/m^3^s) |
| --- | --- | --- | --- | --- | --- | --- | --- |
| Skin | 1.07∙10^-7^ | 0.41 | 1.5 | 10 | 10 | 44 | 0.76 |
| Fat | 1.215∙10^-7^ | 0.3 | 36.5 | 1 | 12 | 45 | 0.22 |

**References**

1. Milanic, M., Muc, B. T., Jezersek, M. & Lukac, M. Experimental and numerical assessment of hyperthermic laser lipolysis with 1,064 nm Nd:YAG laser on a porcine fatty tissue model. *Lasers Surg. Med.* **50**, 125–136 (2018).

2. Milanic, M., Muc, B. T., Lukac, N. & Lukac, M. Numerical Study of Hyper-Thermic Laser Lipolysis With 1,064 nm Nd:YAG Laser in Human Subjects. *Lasers Surg. Med.* **51**, 897–909 (2019).

3. Song, C. W., Park, H. J., Lee, C. K. & Griffin, R. Implications of increased tumor blood flow and oxygenation caused by mild temperature hyperthermia in tumor treatment. *Int. J. Hyperth.* **21**, 761–767 (2005).

4. Pennes, H. H. Analysis of Tissue and Arterid Blood Temperatwes. *J. Appl. Phys.* **1**, 20 (1948).

5. Dřždǎl, T., Togni, P., Víšek, L. & Vrba, J. Comparison of constant and temperature dependent blood perfusion in temperature prediction for superficial hyperthermia. *Radioengineering* **19**, 281–289 (2010).

6. Hickson, R. I., Barry, S. I., Mercer, G. N. & Sidhu, H. S. Finite difference schemes for multilayer diffusion. *Math. Comput. Model.* **54**, 210–220 (2011).

7. Melo, A. R., Loureiro, M. M. S. & Loureiro, F. Blood Perfusion Parameter Estimation in Tumors by means of a Genetic Algorithm. *Procedia Comput. Sci.* **108**, 1384–1393 (2017).
